# Current advances in diabetic neuropathy: Proteins as therapeutic targets

**DOI:** 10.1016/j.isci.2025.113466

**Published:** 2025-09-01

**Authors:** Gurjeet Kaur, Salaha Osman Ali, Alberto Santos, Tina Okdahl, Anne-Marie Wegeberg, Tarunveer S. Ahluwalia, Christian Stevns Hansen, David Wishart, Nicolai J. Wewer Albrechtsen, Birgitte Brock, Troels Staehelin Jensen, Peter Rossing, Christina Brock, Cristina Legido-Quigley, Karolina Sulek

**Affiliations:** 1 Steno Diabetes Center Copenhagen, Clinical Research, Translational Type 1 Diabetes, Translational Medicine, Herlev, Denmark; 2 Institute of Pharmaceutical Science, School of Life Science & Medicine, King’s College London, London, UK; 3 Novo Nordisk Foundation Center for Biosustainability, Technical University of Denmark, Kongens Lyngby, Denmark; 4 Li-Ka Shing Big Data Institute, University of Oxford, Oxford, UK; 5 Mech-Sense, Department of Gastroenterology and Hepatology, Aalborg University Hospital and Department of Clinical Medicine, Aalborg University, Aalborg, Denmark; 6 The Bioinformatics Center, Department of Biology, University of Copenhagen, Copenhagen, Denmark; 7 University of Alberta, Edmonton, AB, Canada; 8 Novo Nordisk Foundation Center for Protein Research, University of Copenhagen, Copenhagen, Denmark; 9 Department of Clinical Biochemistry, University Hospital Copenhagen – Bispebjerg, Copenhagen, Denmark; 10 Danish Pain Research Centre, Department of Clinical Medicine, Aarhus University, Aarhus, Denmark; 11 Department of Clinical Medicine, University of Copenhagen, Copenhagen, Denmark

**Keywords:** Endocrinology, Pathology, Human metabolism

## Abstract

Diabetic neuropathy is a debilitating complication of diabetes characterized by nerve damage that may lead to numbness, foot ulcers, and amputations, and is a major cause of morbidity and mortality in people with diabetes. There are no treatments available for diabetic neuropathy aside from pain management. Recent advances in protein research have identified potential targets associated with the disease development and progression. This review explores the latest studies identifying proteins as possible drug targets in diabetic neuropathy and their role in relevant processes such as polyol metabolism, oxidative stress, and cytokine regulation. Additionally, we provide a comprehensive view of current developments and discoveries at single cell and spatial resolution that have revealed deregulated protein profiles in the dorsal root ganglia, sciatic nerve, trigeminal ganglion, or Schwann cells. Finally, we discuss the benefits of proteomics technologies to identify proteins and associated signaling pathways to better understand the source of diabetic neuropathy.

## Graphical abstract

**Figure:**
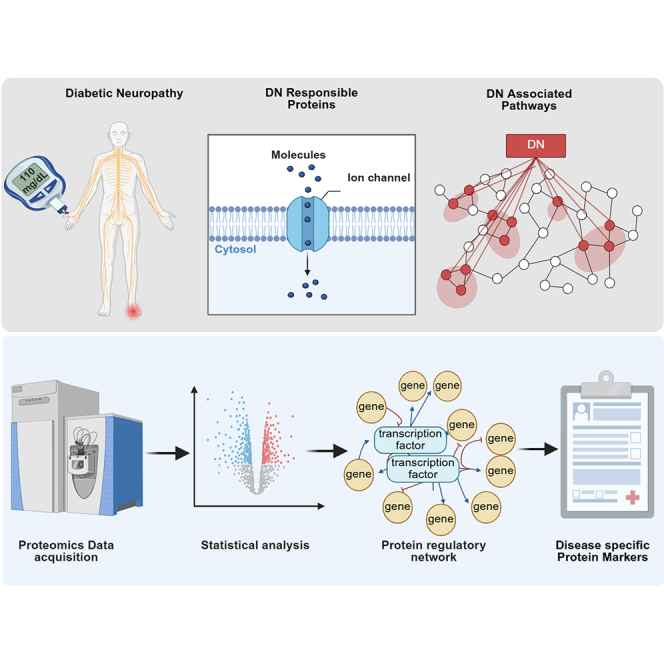

## Introduction

Diabetes is a chronic metabolic disorder characterized by hyperglycemia alongside abnormal lipid and protein metabolism. It is a major global health issue affecting around 530 million people worldwide^1^ The number of diabetes cases is expected to rise to 643 million by 2030 Type 1 Diabetes Mellitus (T1D) is an insulin deficiency caused by the autoimmune and idiopathic destruction of B-cells, while generally Type 2 Diabetes Mellitus (T2D) is a combination of insulin resistance and metabolic syndrome, resulting in limited capability to metabolize glucose.^2^ The cause of T1D is still unknown, although there might be a genetic component. In contrast, T2D risk has been linked to ethnicity and lifestyle factors such as diet and exercise. Several genes have been linked to diabetic neuropathy, but only two, ACE (angiotensin-converting enzyme) and MTHFR (methylenetetrahydrofolate reductase) polymorphisms, have been studied in large cohorts.^3^^,^^4^ Both T1D and T2D are associated with macrovascular and microvascular complications, with diabetic neuropathy (DN) accounting for up to 50% of microvascular complications.^5^ Damage to the peripheral nervous system in diabetes affects sensory, autonomic, and motor axons.^2^ DN is strongly linked with morbidity and mortality in people with diabetes, and there is currently no cure for this condition.^6^ Major known predictors for DN are Hemoglobin A1c (HbA1c) levels, a test of glycated hemoglobin, which reflects average daily glucose levels, and the duration of diabetes.^7^^,^^8^

This review evaluates the potential of protein targets for the prevention and treatment of DN. Proteomics is assessed as a modern tool for disease profiling in search for pathways, which could potentially be altered through medical interventions.^9^ We discuss the current understanding of peripheral nerve injury in animal models and show the disconnect and lack of translatability of these studies compared with the symptoms and phenotypes seen in people with diabetes. Considering the recognized knowledge gap between these basic science results and clinical translation, we have presented an outline of both science strategies to bridge this gap.

This review provides a comprehensive view of DN and the current knowledge regarding proteins and pathways, and their relationship with diabetic neuropathy and their therapeutic potential.

### Diabetic neuropathy

#### Prevalence and symptoms

Nephropathy, retinopathy, and neuropathy are all chronic microvascular complications of diabetes caused by excessive glucose level variations in the blood and other metabolic factors, which result in protein glycosylation and osmotic cell injury, thereby accelerating atherosclerosis. The symptoms and signs of distal symmetric polyneuropathy (DSPN) are characterized by either negative phenomena with loss of numbness and loss of sensation to temperature, touch, pinprick, vibration, or positive phenomena with pain, tingling, or burning sensations. In addition to sensory neuropathy there may also be signs of damage to the motor and autonomic nervous system.^10^

#### Current understanding of DN pathogenesis

Hyperglycemia-induced oxidative stress interferes with polyol metabolism, resulting in excessive superoxide production, elevated reactive oxygen species (ROS), and numerous downstream pathogenic processes.^11^ Long-term hyperglycaemia also predisposes a diabetic nerve to vascular injury, impairing its ability to receive essential nutrients.^11^ Substantial experimental evidence indicates that diabetes attacks the neuron, from the perikaryon to the terminal. The order in which peripheral axons and their associated Schwann cells, or the neuron perikarya that reside in the dorsal root ganglia (DRG) and sustain axons, are damaged is still debatable. Neurons damaged by hyperglycemia and oxidative stress, leading to the activation of TLR-4 and NF-κB pathways in microglia. Activated microglia release pro-inflammatory cytokines and ROS, exacerbating neuronal damage and creating a chronic inflammatory state. Astrocytes, while attempting to support neurons, may become dysfunctional and contribute to the pathology through the impaired regulation of glutamate and neurotrophic factors.^12^ This vicious cycle of damage and inflammation underlies the progressive nature of diabetic neuropathy. Cytokines can stimulate the synthesis of prostaglandins, which cause inflammation and pain, interact with pain-sensing neurons or nociceptors in the nervous system, and modulate neurotransmitters and other signaling molecules involved in pain transmission. DN is a multifactorial disease, and understanding these mechanisms will contribute to the development of effective therapeutic strategies for diabetic neuropathy. Pharmacological agents such as tricyclic antidepressants, selective serotonin and noradrenaline reuptake inhibitors, and gabapentinoids are commonly used for treating the pain associated with DN.^11^ Most pharmacological agents used in clinical settings target the symptom of pain in DSPN rather than the nerve damage, and long-term use of opioids may lead to physical dependence, overdose, and death.^13^ In summary, alternative therapies are needed to treat DN nerve degeneration and related pain effectively.^14^

### Protein alterations in diabetic neuropathy

#### Oxidative stress

**Figure 1:**
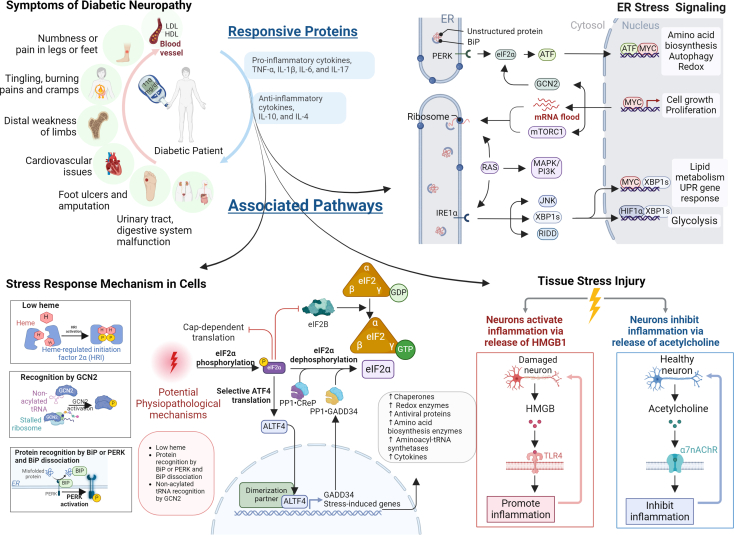
Symptoms of diabetic neuropathy, the responsive proteins involved, and associated pathways, including stress response mechanisms in cells, ER stress signaling, and tissue stress injury Each panel highlights the interplay between these factors in the development and progression of diabetic neuropathy.

Oxidative stress is a phenomenon that occurs due to an imbalance between the production of reactive oxygen species (ROS) and the antioxidant system. This affects the expression and function of several proteins that play a significant role in the development of DN (Figure 1). Chronic exposure to elevated ROS is implicated in the pathogenesis of various diseases, highlighting the critical role of oxidative stress in cellular damage and disease progression. Elevated ROS often begin with increased production from mitochondria, NADPH oxidases, or other sources under stress conditions. ROS, including superoxide anion (O2·−), hydrogen peroxide (H2O2), and hydroxyl radical (·OH), disrupt cellular homeostasis by oxidizing lipids, proteins, and DNA. This oxidative stress triggers signaling pathways that promote inflammation, alter gene expression, and contribute to cellular dysfunction. ROS plays a critical role in the impaired mitochondrial function, activated cell death, axonal transport, and impaired neurovascular function. These mechanisms collectively contribute to the progressive deterioration of nerve function observed in diabetic neuropathy.^15^ The oxidative stress and peripheral nerve damage in muscle creatine kinase promoter/human insulin growth factor 1 (IGF-I) receptor in muscle creatine kinase promoter (MKR) T2D mice were closely associated with increased levels of cytochrome-P450 (CYP4A) and 20-Hydroxyeicosatetraenoic acid (20-HETE) (Table 1).^16^ The study showed that NADPH oxidase played a role in the DN animal model by linking CYP4A/20-HETE to enhanced ROS generation via an NADPH-dependent route.^16^ Additionally, autophagy dysregulation was identified as a potential cause of DN, as evidenced by increased Beclin-1 and LC3B protein expression. N-Hydroxy-N′-(4-butyl-2-methylphenyl)-formamidine (HET0016), a CYP4A inhibitor, was found to normalize autophagy protein markers and activate AMP-activated protein kinase (AMPK), which reduces NADPH-produced ROS in diabetic rats, suggesting its involvement in providing neuroprotection by targeting AMPK.^17^ Although studies have investigated T1D and T2D models, as well as NADPH expression in streptozotocin (STZ) and MKR-induced mice, respectively, however, the findings need further validation in people with diabetes.^16^^,^^17^

**Table 1:** This table provides a comprehensive overview of key proteins involved in diabetic neuropathy, their regulatory pathways, physiological outcomes, model systems used, and translational potential

Protein name	Up/Down regulated	Regulatory pathway targeted	Physiological outcome	Model used	Translation (Y/N)	Reference
Insulin growth factor 1 (IGF-I)	Up	Oxidative stress signaling pathway	Increases the level of cytochrome-P450 (CYP4A) and 20-Hydroxyeicosatetraenoic acid (20-HETE)	DN rats	N	15
Beclin-1 and Microtubule-associated proteins 1A/1B light chain 3B (LC3B)	Up	AMP-activated protein kinase (AMPK)	Involvement in providing neuroprotection by targeting AMPK	MKR T2D Mice	N	16
Adaptor protein motif 1 (APPL1)	Down	Mammalian target of rapamycin (mTOR)	The downregulation of APPL1 in PDN rats increased mTOR activation in the spinal cord, which exacerbated mechanical hyperalgesia	PDN rats	N	17
Interleukin (IL)-1β, IL-6, IL-17, and C-reactive protein	Up	p38- Mitogen-activated protein kinase (MAPK) upstream kinase activator	Strategies focusing on reducing oxidative stress, modulating autophagy, activating AMPK, and inhibiting inflammatory pathways may prove effective in managing DN.	Patients with DN vs. control	N	21, 22
Tumor Necrosis Factor alpha (TNF-α)	Up	TNF-α signaling pathway	TNF antagonists have the potential to alleviate neuropathic pain	Patients with subacute lumbosacral radiculopathy vs. the control group	N	24, 25
IL-6, TNF, and High-mobility group box 1 (HMGB1)	Up	Nuclear factor kappa B (NF-κB) signaling	Stress and inflammatory signaling pathways	patients with DN vs. control	N	35
Chemokine (C-C motif) ligand 1 (CCL1) and Ionized calcium-binding adaptor molecule 1 (IBA1)	Up	CCL1/CC chemokine receptor 8 (CCR8) signaling	Induced hypersensitivity and caused microglia to release IL-1 and IL-6, pronociceptive cytokines	STZ-induced diabetic rats	N	37
IL-4	Up	Anti-inflammatory signaling	IL-4 intrathecal injections reduce inflammation and mechanical allodynia in animal models of nerve injury	Rats with partial nerve ligation	N	38
Transforming growth factor β (TGF-β1)	Up	TGF-β signaling	Maintaining blood-spinal cord barrier integrity and regulating extracellular matrix synthesis and cross-linking.	DN Rats	N	43
Aldose reductase (AR)	Down	Polyol pathway	Long-term treatment with epalrestat is well tolerated and can effectively delay the progression of DN.	patients with DN Epalrestat group and control group	Y	49
Mitochondrial calcium uniporter (MCU)	Down	Calcium signaling	Blocking calcium entry through MCU can restore normal mitochondrial function and alleviate neuropathic symptoms	PDN mice	N	55
Mitogen-activated protein kinase 1 (MAPK1)	Down	MAPK/extracellular signal-regulated kinase (ERK) signaling pathway	High glucose-induced demyelination and impaired nerve function, suggesting its involvement in diabetic neuropathy	Schwann cells	N	55
Cysteine and glycine-rich protein 1 (CSRP1)	Down	Spinal cord regeneration pathways	Impaired nerve regeneration and repair	Spinal cord injury models, diabetic neuropathy models	N	55
Tenascin-R (TNR)	Up	TNR pathway	DRG neuron degeneration impedes axonal regeneration	patients with DN vs. controls	N	56
Thioredoxin 2 (Trx2)	Up	Mitochondrial related pathway	Improves the myelin sheath and peripheral nerve function in diabetic neuropathy	Schwann cells	N	57

In painful diabetic neuropathy (PDN) rats, lower levels of adaptor protein motif 1 (APPL1) protein were observed in the lower spinal cord, and laminae I and II, when compared to controls. The downregulation of APPL1 in PDN rats increased mTOR activation in the spinal cord, which exacerbated mechanical hyperalgesia.^18^

In a study involving 40 healthy subjects and 28 patients with DN, significant differences (*p* < 0.05) were observed in the antioxidant indicators between the two groups, while no significant differences were observed in total protein (TP), Globulin, Albumin, albumin/globulin ratio (AGR), Thiol, and free amine between healthy subjects and patients with DN.^19^

Furthermore, the accumulation of advanced glycation end products (AGEs) and oxidation end products (OPs) contributes to the deterioration of the vascular wall inT1D.^20^ The AGE/receptor for AGEs (RAGE) interaction may link microangiopathy to DN. AGE build-up promotes the production of proinflammatory cytokines and adhesion molecules such as E-selectin, IL-6, TNF, and VEGF, which leads to vascular endothelial dysfunction in arteries of different diameters, and might serve as biomarkers of DN. In addition, higher levels of serum NFL were associated with DSPN, which may aid in the diagnosis and treatment of DSPN in diabetes with recent onset.^21^

#### Pro-inflammatory cytokines in oxidative stress

The pro-inflammatory cytokines TNF-α, IL-1β, IL-6, and IL-17 are up-regulated and expressed through the p38-MAPK upstream kinase activator. In addition, these cytokines are also significantly elevated in cerebrospinal fluid (CSF) and blood of patients with long-term neuropathic pain.^22^^,^^23^ People with diabetes have an increased expression of pro-inflammatory cytokines, such as C-reactive protein, TNF-α, and IL-6.^24^ These findings suggest that the pro-inflammatory cytokines play a crucial role in the pathogenesis of DN-induced pain and may serve as potential therapeutic targets in the management of neuropathic pain. (Figure 1).

#### Tumor necrosis factor alpha

Pro-inflammatory cytokine TNF-α plays a crucial role in the emergence of DN. Its plasma concentration and the number of macrophages have been found positively correlated with the progression of DN.^25^^,^^26^ A placebo-controlled, dose-response study found that the transforaminal epidural injection of Etanercept, a TNF inhibitor, significantly reduced long-term leg pain in patients with subacute lumbosacral radiculopathy compared to the control saline group.^27^ TNF antagonists have the potential to alleviate neuropathic pain, however, further research is needed to establish their therapeutic effectiveness in a larger population.

#### Interleukin-1, Interleukin-6, and Interleukin-17

IL-1 is a proinflammatory cytokine that plays a critical role in initiating the host's inflammatory and immunological responses. In the context of peripheral nerve injury, diabetic rat models of neuropathy exhibit elevated spinal IL-1 in various cells.^28^ Furthermore, painful peripheral neuropathies have higher IL-1 levels in CSF than healthy controls.^29^ Studies have demonstrated that suppressing caspase 1, which regulates IL-1 maturation, reduces mechanical allodynia and thermal hyperalgesia after constriction injury (CCI), confirming the involvement of IL-1 in nerve pain.^30^ Interleukin-6 is an inflammatory cytokine with wide-ranging biological effects. Although IL-6 plays vital roles in host defense and homeostasis maintenance, it has also been widely demonstrated that neuroinflammation plays a critical role in the development of pathological pain.^31^

Prostaglandin E2 (PGE2) is a lipid mediator that activates EP4 receptors, leading to the production of IL-6 in DRG neurons after partial sciatic nerve ligation.^32^ The protein kinase (PKC) pathway and EP4 receptor in invading macrophages through PGE2 suggests a potential role for PGE2 in the induction of IL-6 in neuropathic pain. Studies have shown that reducing IL-6 expression can alleviate pain in animal models of neuropathy.

Tocilizumab, an IL-6 receptor inhibitor, has shown promise in treating sciatica and discogenic low back pain in clinical studies. However, elevated IL-6 levels have been observed in regions beyond the site of nerve injury, indicating that IL-6 may be a non-specific marker of neuroinflammation.^33^ In a study by Sainoh et al., intradiscal injections of Tocilizumab with Bupivacaine were compared to Bupivacaine alone for discogenic back pain and found statistically significant short-term pain relief and improvement at 2 and 4 weeks, but not at 6 weeks.^34^ However, Dubový et al. demonstrated that elevated IL-6 levels were not only observed in the injured nerve but also on the opposite side and in cervical regions following unilateral lumbar CCI in rats.^35^ This suggests that IL-6 or other cytokines may serve as non-specific markers of neuroinflammation. Further research is needed to fully understand the role of IL-6 and cytokines in neuropathic pain and explore their potential as therapeutic targets.

#### Caspase 3 and p38MAPK

In DN, several inflammatory cytokines have been implicated in the development of neuropathic pain. An *in vitro* study has validated Caspase 3, IL-6, IL-1, and p38MAPK as DN targets.^36^ In patients with T2DM, IL-6, TNF, and HMGB1 levels were found to be considerably higher than in controls. These results suggest that hyperglycemia elevated HMGB1 and nuclear factor kappa B (NF-κB) signaling, which boosted pro-inflammatory cytokines *in vivo* and *in vitro*. Further, experimental evidence has shown expression of HMGB1 in the sciatic nerves of diabetic rats.^37^ Active p38MAPK modulates stress and inflammatory signaling pathways, which causes DN.

#### CCL1 and IBA1

CCL1 has also been implicated in the development of neuropathic pain in STZ-induced diabetic rats. On day 7, an increase in CCL1 (34%) and IBA1 (40%) was observed, which induced hypersensitivity and caused microglia to release IL-1 and IL-6, pronociceptive cytokines.^38^ Neutralizing CCL1 with antibodies reduced pain behavior on day 7 following STZ, confirming its role in neuropathic pain. CCR8, a receptor protein necessary for pronociceptive CCL1, did not increase in STZ-induced animals, implying that the turnover of CCR8 may be increased in neuropathy.^38^ The findings suggest that CCL1 changes neuroimmune interactions, and that CCL1/CCR8 signaling enhances DN pain.

In conclusion, IL-17, Caspase 3, IL-6, IL-1, p38MAPK, HMGB1, CCL1, and IBA1 have all been implicated in the onset and maintenance of neuropathic pain. While research on these targets is still ongoing, they hold promise as potential therapeutic targets for the treatment of neuropathic pain.

#### Anti-inflammatory cytokines

Neuropathy is often associated with chronic inflammation, which makes anti-inflammatory cytokines an attractive therapeutic target for managing the DN. Despite the promising results seen in animal models and *in vitro* studies, more research is needed to fully understand the role of anti-inflammatory cytokines in DN and related pain. Future studies should explore the potential benefits and risks of anti-inflammatory cytokine therapy in humans and investigate the optimal dose and duration of treatment for maximum therapeutic effect.

#### Interleukin-4, interleukin-10, and transforming growth factor β

IL-4, a well-known anti-inflammatory cytokine, is an essential immune regulator that is released by various immune cells. Studies have shown that IL-4 intrathecal injections can decrease inflammation and pro-inflammatory cytokine levels in animals with CCI and reduce mechanical allodynia in rats with partial nerve ligation.^39^ Furthermore, the pain-relieving actions of Glatiramer acetate, a medication used for treating multiple sclerosis, are linked to an increase in two anti-inflammatory cytokines, IL-4 and IL-10, in the spinal cord.^40^

IL-10 is a cytokine that functions as an anti-inflammatory agent by inhibiting the production of pro-inflammatory cytokines through the decrease of nuclear factor-κB (NF-κB) activity. Several studies have reported lower levels of IL-10 in the CSF of patients affected by neuropathy compared with healthy controls, and a negative correlation between IL-10 levels and their pain ratings.^41^^,^^42^ Interestingly, Glatiramer acetate therapy has been shown to reverse neuropathic hypersensitivity and coincides with higher IL-10 expression levels in both T cells and other cells in the spinal cord.^43^ This suggests that manipulating the balance of Th1 and Th2 cells in the spinal cord, which stimulate the immune response, could be a potential approach to treat neuropathy, yet only from the pain management side.

In recent years, there has been increasing interest in the role of the TGF-β1 in diabetes complications. Interestingly, previous studies have shown that TGF-β infusion can alleviate pain induced by partial nerve ligation and CCI.^44^ Conversely, the injection of anti-TGF-β antibodies into the red nucleus promotes mechanical hypersensitivity in rats (Table 1).^44^ TGF-β1 therapy has been shown to sustain higher levels of tight junction proteins following nerve injury, thus maintaining the blood spinal cord barrier integrity in rats.^45^ In addition, the renal expression of TGF-β1 mRNA and protein is increased in patients with diabetes mellitus, and it enhances the synthesis and cross-linking of extracellular matrix (ECM). Flexibilide has been demonstrated to alleviate neuropathic pain in rats by preventing TGF-β decrease following CCI. Furthermore, infusion of bone marrow stromal cells into the spinal cord of rats has been used to boost TGF-β production, resulting in a reduction in CCI-induced neuropathic pain.^46^ These findings suggest that targeting TGF-β signaling may hold promise as a potential therapeutic strategy for the treatment of diabetic neuropathic pain.

#### Proteins in polyol metabolism

Polyol metabolism has been identified as a major cause of DN, which is characterized by a range of metabolic imbalances resulting from hyperactivity in the metabolic pathways that process and degrade polyols or sugars. This pathway involves the reduction of glucose to sorbitol by the enzyme aldose reductase (AR), and the subsequent conversion of sorbitol to fructose by sorbitol dehydrogenase. Under normal glucose levels, the polyol pathway accounts for a small fraction of glucose metabolism. However, in hyperglycemic conditions typical of diabetes, excess glucose enters the polyol pathway, leading to several detrimental effects. This process depletes NADPH, reducing the cell’s ability to counteract oxidative stress, and increases intracellular osmotic stress, leading to nerve damage. Moreover, the accumulation of sorbitol disrupts Na+/K+ ATPase activity, impairing nerve function, while also promoting oxidative stress and activating protein kinase C, further damaging nerves. Inhibiting aldose reductase has been explored as a therapeutic approach but has shown limited clinical success.^47^ Overall, the promising potential of this pathway has been interpreted in various studies in different ways. While some AR inhibitors showed negative effects in the drug trials, where animal results could not be replicated in human studies,^48^ epalrestat has been shown to delay the progression of diabetic neuropathy and reduce the associated symptoms of the DN.^49^^,^^50^ In addition, advanced glycation end-products (AGEs) produced from the polyol pathway, such as glucoselysine, arise from the non-enzymatic glycation of proteins and lipids due to elevated fructose levels. This process is heightened in hyperglycemic conditions, leading to increased oxidative stress and inflammation. AGEs such as glucoselysine have been associated with vascular complications in type 2 diabetes, serving as potential biomarkers for monitoring disease progression and therapeutic efficacy. Understanding these AGEs is crucial for addressing diabetes-related complications and developing targeted interventions.^51^

#### Neuronal regeneration

Growth factors play a vital role in the development and maintenance of the nervous system, including nerve growth and regeneration. Studies have shown that growth associated protein 43 (GAP43) in immunoreactive nerve fibers is reduced in diabetics.^52^ It has been suggested that the expression of GAP43 may be linked to axon regeneration and remodeling, indicating that reduced expression in skin nerves may be an early indicator of diabetic neuropathy and a potential therapeutic target for the condition.^52^ Further studies are needed to determine the normal range for GAP43 immunoreactive intraepidermal nerve fibers and to expand the sample size to test this hypothesis. Axonal regeneration-inhibitory molecules, such as RhoA and PTEN, play a significant role in the development of DN by hindering nerve repair mechanisms. RhoA disrupts cytoskeletal dynamics, preventing axonal growth, while PTEN suppresses the PI3K/Akt pathway, which is essential for neuronal survival and regeneration. Both molecules, through distinct but overlapping mechanisms, create a regeneration-inhibitory environment that exacerbates neuropathic symptoms in diabetes.^53^^,^^54^

### Proteomics applications in diabetic neuropathy

**Figure 2:**
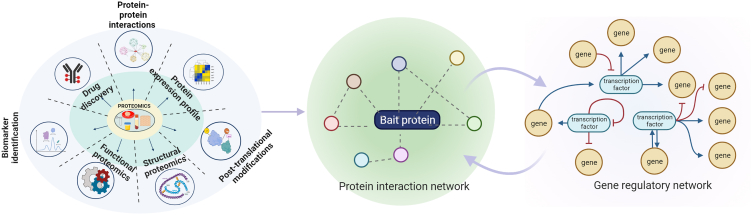
Applications of proteomics in clinical research, showcasing three types of proteomic approaches and their potential impact Systems biology approaches are used to study protein-protein interactions and signaling pathways, facilitating a better understanding of disease mechanisms and identification of potential drug targets. Regulatory network analysis enables the investigation of transcriptional and post-transcriptional regulation of protein expression, facilitating the discovery of disease-associated regulatory pathways and mechanisms.

Proteomics has emerged as a promising technology in clinical practice, offering a valuable strategy for exploring the pathogenesis of various diseases, including diabetes mellitus and related complications.^55^ Proteomics is a rapidly advancing field that is expected to revolutionize drug development in the coming years. It enables the identification of potential drug targets by detecting differentially expressed proteins in healthy and diseased individuals, which can be tested against chemical compound libraries to identify lead compounds that could serve as new therapies.^55^ The development of better diagnostic tools and treatments using proteomics knowledge is the next opportunity (Figure 2).

**Figure 3:**
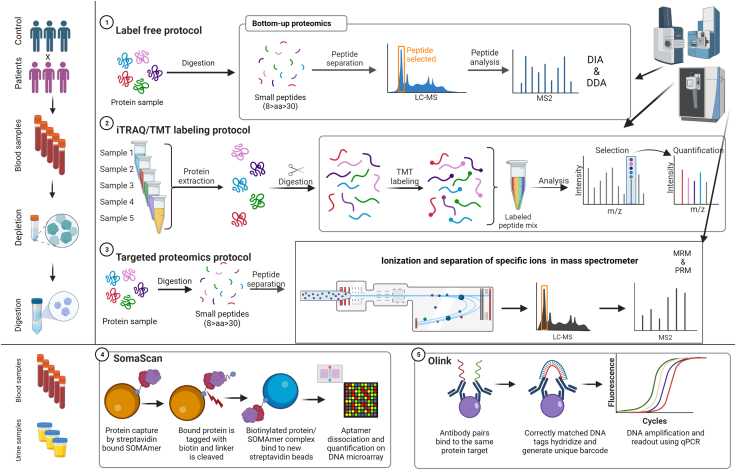
Schematic representation of various clinical blood sample-based proteomics approaches for the quantification and characterization of protein biomarkers The figure outlines four different proteomics protocols, including label-free quantification, iTRAQ/TMT labeling, targeted proteomics, and two commercially available platforms, SomaScan and Olink, for high-throughput protein analysis. The label-free quantification approach involves the direct comparison of protein abundance between two or more samples based on their spectral intensity. The iTRAQ/TMT labeling protocol involves labeling of peptides with isotopic tags, allowing for the relative quantification of proteins. The targeted proteomics approach involves the selective identification and quantification of specific protein targets using mass spectrometry. The SomaScan and Olink platforms are both based on multiplexed immunoassays, allowing for the simultaneous analysis of many protein targets in a single sample. SomaScan uses aptamer-based technology, while Olink employs proximity extension assay (PEA) technology. In summary, these proteomics approaches offer powerful tools for the discovery and validation of protein biomarkers in clinical blood samples, with potential applications in disease diagnosis, prognosis, and monitoring.

Mass spectrometry (MS) technology has played a crucial role in enabling the precise identification and high-throughput analysis of proteins on a large scale. This technology has contributed significantly to unraveling key protein-protein interactions, discovering signaling networks, and understanding disease mechanisms.^56^ Bottom-up proteomics, which involves the proteolytic digestion of proteins extracted from any biological sample, followed by the liquid chromatography (LC) separation of the resulting peptides, is the most used approach. The peptides are then eluted and subjected to electrospray ionization (ESI), which allows for the precise identification of amino acid sequences of the peptides. The peptide sequences can then be mapped to infer the proteins using computational algorithms (Figure 3).

Bottom-up proteomics can also offer quantitative analysis, allowing for the assessment of relative changes in protein abundance.^57^ Label-free quantification (LFQ) is a popular method that uses ion signal intensities to quantify protein abundance. Although LFQ offers several advantages, including simple sample preparation, the ability to analyze a multitude of peptides, and unlimited conditions to be analyzed, challenges related to reproducibility and sample handling must be overcome. Strategies based on chemical labeling, such as isobaric tags, have gained attention due to their ease and speed of use. These methods allow for multiplexing, which has reduced technical variability. Isobaric labels such as iTRAQ and TMT are widely utilized to quantify proteins reliably and answer diverse biological questions of clinical relevance. Targeted proteomics is another emerging technique that enables the detection and quantification of specific proteins of interest in a highly multiplexed manner.^58^ (Figure 3).

While immunoassays have been widely used for the quantitative analysis of plasma proteins in clinical diagnostics, they have inherent limitations in terms of multiplexing, specificity is not optimal, particularly for protein isoforms, and compatibility with hypothesis-free investigations. In contrast, mass spectrometry (MS)-based proteomics has the potential to overcome these limitations, where isoforms are detected through tandem mass spectrometry and omics-based profiling is not limited to a targeted approach with a limited number of proteins. Hence, omics application could be the optimal tool to identify protein targets in tissue biopsies as well as easily accessible body fluids such as blood.^59^

### Advance in proteomics in relation to diabetic neuropathy

**Figure 4:**
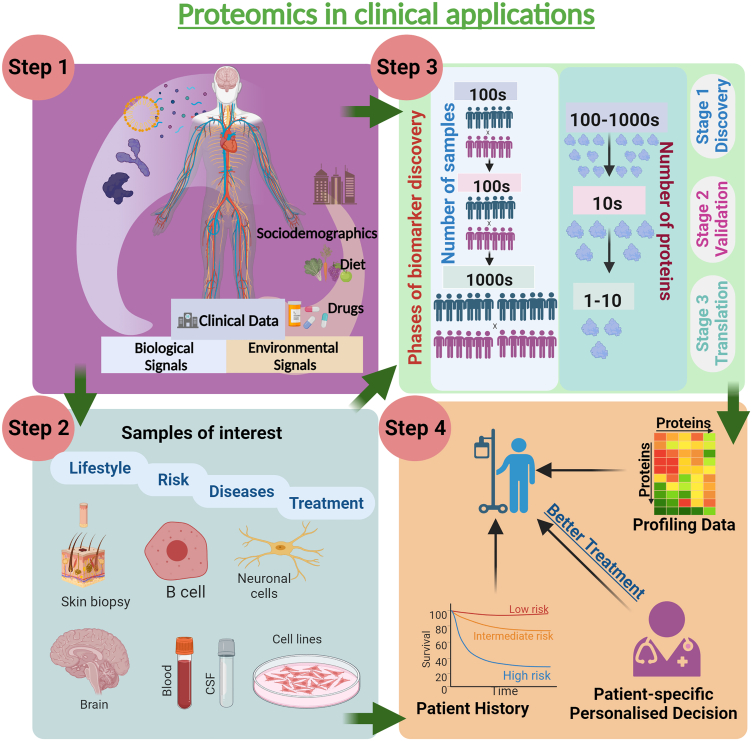
Clinical applications of proteomics in clinical research, showcasing four steps of biomarker discovery and implementation in clinical settings for a comprehensive and nuanced understanding of disease processes, paving the way for personalized medicine and targeted therapies

Most of the research in this proteomic literature review focuses on changes in protein localization in the DRG, Sciatic Nerve (SN), and Trigeminal Ganglion (TG). A total of 683 of 2,356 proteins (28.9%) in the SN showed significant changes in diabetes, with Ingenuity Pathway Analysis (IPA) discovering dysregulation in oxidative phosphorylation, LXR/RXR activation, and glycolysis, while increasing glucose levels had little effect on total protein expression. A recent study reported 85 (5.2%) of the 1,649 DRG proteins and 60 (3.4%) of the 1,734 TG proteins were differentially expressed.^60^ Proteomics can also be utilized to better understand disease-related mechanisms and discover biomarkers, utilizing model systems, animal models, and human samples that may act as diagnostic markers or therapeutic targets, which could improve DN treatment (Figure 4).

### Sciatic nerve, dorsal root, and Schwann cells

In recent years, there has been increasing interest in the role of lipid metabolism in neuropathy, and it has been suggested that dysfunctional lipid metabolism may be one of the causes of neuropathy in the SN.

Peripheral sensory neurons that conduct pain have cell bodies in the DRG, making it a useful tool for studying molecular pathways and medications in Schwann cell (SC) growth and myelination. The link between DRG and DN has been established, with DN characterized by DRG nociceptor hyperexcitability, neuropathic pain, calcium overload, axonal deterioration, and cutaneous innervation loss, although the underlying molecular pathways remain unknown. Studies have shown that DPN elevates immune-related gene expression while lowering neuronal gene expression in human DRG. Proteomic changes in high-fat diet (HFD) diabetic rodent DRG suggest altered energy metabolism and mitochondrial function, including increased DRG nociceptor calcium signaling, the phosphorylation of RNA-binding proteins, and structural alterations of extracellular matrix proteins.^60^

In a study involving neurons generated from DRG of diabetic rats maintained in culture with high and normal glucose conditions, proteins associated with oxidative phosphorylation and the tricarboxylic cycle were downregulated in the sural nerve and spinal nucleus but not in the DRG in diabetes.^60^ Proteome analysis of lumbar DRG extracts from regular-fed or HFD-fed mice for 10 weeks identified 5249 proteins in 8 biological replicates from each group. A longitudinal proteomic study identified 247 proteins out of the 6186 in the DRG as differentially expressed using mass spectrometry.^61^ Another 10-week study identified 1121 differentially expressed proteins between regular diet (RD) and HFD fed mice, including numerous pathways, but did not mention the total number of proteins expressed. However, larger cohorts with longitudinal changes are necessary to identify differentially expressed proteins to dissect the complexity of the disease.

Recent research has focused on understanding mitochondrial dysfunction in the development of diabetic neuropathy. Studies have shown that in mice on HFD, there is an upregulation of mitochondrial proteins associated with fission, such as Fission 1 protein (Fis1), Dynamin-1-like protein (Drp1), and Mff, in the lumbar DRGs.^61^ Morphological studies have revealed fragmented mitochondrial structures in HFD DRGs before the onset of neuropathic symptoms. Calcium dysregulation is also observed, with HFD DRG nociceptors displaying larger calcium transients in response to mechanical stimuli. The mitochondrial calcium uniporter (MCU) has been identified as a potential therapeutic target for DPN treatment, as blocking calcium entry through MCU can restore normal mitochondrial function and alleviate neuropathic symptoms. Additionally, calcium signaling plays a role in regulating Drp1 phosphorylation and mitochondrial dynamics. Reduced respiratory chain proteins and activity have been observed in DRG neurons of mice with type 2 diabetes, indicating an influence of DPN on mitochondrial structure and location.^60^ In DPN, blocking the MCU can decrease mitochondrial fission and protect against ischemia/reperfusion damage, which can be induced by atherosclerosis in diabetic neuropathy. Tenascin-R (TNR), a protein associated with DRG neuron degeneration, has been found to impede axonal regeneration. Patients with DPN exhibit higher levels of TNR surrounding DRG neurons compared to control subjects, suggesting its potential role in the development of PDN. Targeting the TNR pathway may be considered in future treatments for PDN.^61^

A study by Chong et al. showed that short-term exposure of human Schwann cells (HSC) to varying glucose concentrations did not show significant effects on SC proliferation, cytotoxicity, apoptosis, and sPLA2 expression. However, long-term exposure to high glucose levels resulted in reduced expression of mitogen-activated protein kinase 1 (MAPK1) and cysteine and glycine-rich protein 1 (CSRP1), which have been associated with SC myelination and spinal cord regeneration, respectively. This downregulation of MAPK1 may contribute to high glucose-induced demyelination, suggesting its involvement in diabetic neuropathy (DN) (Table 1).

It has been demonstrated that newborn rat derived SCs grown in high glucose conditions exhibited increased oxidative phosphorylation and tricarboxylic acid cycle, which are associated with metabolic and mitochondrial abnormalities in DN.^60^ Excess glucose can lead to metabolic dysfunction in SCs, compromising neuronal functioning and causing neuropathy.

Paeoniflorin (PF) has been found to upregulate the mitochondrial protein thioredoxin 2 (Trx2) in SCs exposed to a high glucose environment.^62^ PF was observed to improve myelin sheath and peripheral nerve function in diabetic neuropathy (DN) rats by enhancing mechanical pain threshold, sensory and motor nerve conduction velocity, and reducing thermal pain threshold. PF achieved this by improving the protein processing of Trx2 in mitochondria and affecting various metabolic pathways.^62^ In addition, Bönhof et al. discovered a positive correlation between ficolin-3, a protein involved in the lectin pathway of the complement system, and diabetic demyelinating neuropathy. This finding suggests the potential involvement of the complement system in the development of DN.

Furthermore, George et al. found that the direct stimulation of DRG neurons with intrathecal insulin increased sensory nerve conduction velocity and protected against distal axonal atrophy and intraepidermal nerve fiber loss in diabetic mice.^60^ Their study also revealed metabolic regulation differences between the cell bodies of the lumbar DRG and the axonal/Schwann cell-rich SN, providing insights into the distribution patterns of peripheral neuropathies. While further research is needed to confirm these findings, these studies highlight the potential for new treatments targeting peripheral nervous system metabolism to alleviate the symptoms of DN.

In addition, Kalteniece et al. identified 12 protein markers that had lower levels in DSPN than in T2D, while the other 5 were higher. The deficiency of growth hormones supporting nerve regeneration and angiogenesis, as well as a complicated interaction between innate and adaptive immunity, may influence the development of DSPN in T2D. In a study, elevated levels of plasma ceruloplasmin, nephropathy, and tear proteins were found in patients with DN, indicating a link to oxidative stress.^63^ Additionally, a decreased expression of von Willebrand protein and complement C3 was observed. While corneal nerve injury was detected, there were no significant variations in previously associated neurotrophins and growth factors.

The nuclear factor erythroid 2 2-related factor 2 (Nrf2) is a molecule involved in regulating the progression of diabetic retinopathy. Chronic hyperglycemia was found to decrease Nrf2 expression, while acute hyperglycemia increased it. The downregulation of Nrf2 is associated with various microvascular changes that contribute to the development of DN. Therefore, the use of Nrf2 activators as a treatment strategy for DN has been suggested.^64^ Furthermore, lower levels of substance P in the tear film have been linked to both corneal changes and painful in T1D with patients with DN, suggesting a potential avenue for future research.

## Limitations of the study

Despite the significant insights gained from recent proteomic analyses in DN, several limitations constrain the translational value and broader applicability of the current findings. First, there remains a major gap between animal models of DN and the clinical phenotypes observed in human patients. While animal models offer valuable insights into molecular mechanisms, their physiological and pathological differences limit direct translation to clinical settings. Second, the proteomic studies reviewed often involve small sample sizes and focus on a limited range of tissues such as DRG, SN, and TG. Although these regions are relevant to DN, the restricted scope may overlook important changes in other components of the peripheral nervous system. Third, although numerous differentially expressed proteins have been identified, their potential as drug target and functional significance remain largely unvalidated in larger human cohorts.

## Future outlook and conclusions

DN is a serious and common complication of diabetes that can cause significant morbidity and mortality. While current pharmacotherapeutic therapies for DN are limited and only reduce pain, the discovery of novel targets is crucial. Several methods for targeting proteins as a treatment for diabetic neuropathy are currently being investigated. One strategy is using cytokines that can influence the activity of specific proteins, such as prostaglandins implicated in the development of neuropathy. In recent years, researchers and diabetic organizations have identified several potential targets, but none have been applied in clinical practice. Hence, larger joint studies with standardized sample collection, processing, and data collection are required for research and validation. Future research should combine an aging population and targets from proteomics, metabolomics, and genomics to generate targets for DN. Collaborative research is required to characterize new targets to develop feasible treatments, standardize methodologies, and estimate costs. To prevent adverse outcomes and improve patient satisfaction, many targets identified in this study need to be validated and analyzed for applicability in clinical practice.

### Search strategy and selection criteria

The objective of this literature review was to identify differentially expressed proteins in diabetic neuropathy and assess their potential as intervention targets or biomarkers. The review searched the Embase (Ovid) database using the keyword combinations “diabetic neuropathy” AND “protein∗” and returned 254 results. To ensure only the most recent and relevant sources were included, 137 sources were excluded based on exclusion criteria that included only primary sources and sources published from 2012 to 2022. From the remaining 110 sources, 61 were irrelevant and 5 were duplicates, leaving 44 sources for the protein component of the review. The search was also completed on Embase (Ovid) using the search terms “diabetic neuropathy AND proteomics,” which yielded an initial 45 results. After applying the same exclusion criteria, 21 primary sources were included in the proteome area of the literature review, with 5 added by free search. In addition, the review focused on cytokines, locating sources using search engines such as Google Scholar, PubMed, and citation searching to confirm original findings with more recent studies. Interestingly, the review found only one out of 1284 studies completed a pharmacological intervention clinical study on DN, which reached phase 4, but has not announced its results since completion in 2018. This illustrates the challenge of discovering pharmaceutical therapies for DN.

## Acknowledgments

All the figures are prepared with the help of purchased BioRender online tool. This research is supported by Novo Nordisk Foundation Excellence Emerging Investigator Grant - Endocrinology and Metabolism 2022 (grant number 0074491 awarded to K.S.): “Omics on call for the prevention of diabetic neuropathy”.

## Author contributions

Conceptualization GK, SOA, CLQ, and KS; writing—original draft preparation GK, SOA, CLQ, and KS, writing—review and editing GK, SOA, ASD, TO, AMW, TVSA, CSH, DW, NJWA, BB,TSJ, PR, CB, CLQ, and KS, project administration CLQ and KS. All the authors have read and agreed to the published version of the article.

## Declaration of interests

PR has received funding from Astra Zeneca, Novo Nordisk, and Bayer, and has received honoraria (to Steno Diabetes Center Copenhagen) for consultancy and teaching from Astra Zeneca, Abbott, Bayer, Boehringer Ingelheim, Gilead, Novo Nordisk, Sanofi. The article was conceptualized and prepared while KS was used at Steno Diabetes Center Copenhagen, and during the final revision, KS has changed affiliation to Novo Nordisk. The other authors disclose no conflicts of interest.
